# Diagnosis of Parkinson syndrome and Lewy-body disease using ^123^I-ioflupane images and a model with image features based on machine learning

**DOI:** 10.1007/s12149-022-01759-z

**Published:** 2022-07-07

**Authors:** Kenichi Nakajima, Shintaro Saito, Zhuoqing Chen, Junji Komatsu, Koji Maruyama, Naoki Shirasaki, Satoru Watanabe, Anri Inaki, Kenjiro Ono, Seigo Kinuya

**Affiliations:** 1 Department of Functional Imaging and Artificial Intelligence, Kanazawa University Graduate School of Advanced Preventive Medical Sciences, 13-1 Takara-machi, Kanazawa, 920-8640 Japan; 2 Department of Nuclear Medicine, Kanazawa University, Kanazawa, Japan; 3 Department of Neurology, Kanazawa University Graduate School of Medical Sciences, Kanazawa, Japan; 4 Wolfram Research Inc., Champaign, IL USA; 5 Department of Chemistry and Materials Science, Osaka City University, Osaka, Japan; 6 Department of Neurosurgery, Kaga Medical Center, Kaga, Japan

**Keywords:** Neurodegenerative disease, Dopamine transporter, Movement disorder, Pattern recognition, Artificial intelligence

## Abstract

**Objectives:**

^123^I-ioflupane has been clinically applied to dopamine transporter imaging and visual interpretation assisted by region-of-interest (ROI)-based parameters. We aimed to build a multivariable model incorporating machine learning (ML) that could accurately differentiate abnormal profiles on ^123^I-ioflupane images and diagnose Parkinson syndrome or disease and dementia with Lewy bodies (PS/PD/DLB).

**Methods:**

We assessed ^123^I-ioflupane images from 239 patients with suspected neurodegenerative diseases or dementia and classified them as having PS/PD/DLB or non-PS/PD/DLB. The image features of high or low uptake (F1), symmetry or asymmetry (F2), and comma- or dot-like patterns of caudate and putamen uptake (F3) were analyzed on 137 images from one hospital for training. Direct judgement of normal or abnormal profiles (F4) was also examined. Machine learning methods included logistic regression (LR), k-nearest neighbors (kNNs), and gradient boosted trees (GBTs) that were assessed using fourfold cross-validation. We generated the following multivariable models for the test database (*n* = 102 from another hospital): Model 1, ROI-based measurements of specific binding ratios and asymmetry indices; Model 2, ML-based judgement of abnormalities (F4); and Model 3, features F1, F2 and F3, plus patient age. Diagnostic accuracy was compared using areas under receiver-operating characteristics curves (AUC).

**Results:**

The AUC was high with all ML methods (0.92–0.96) for high or low uptake. The AUC was the highest for symmetry or asymmetry with the kNN method (AUC 0.75) and the comma-dot feature with the GBT method (AUC 0.94). Based on the test data set, the diagnostic accuracy for a diagnosis of PS/PD/DLB was 0.86 ± 0.04 (SE), 0.87 ± 0.04, and 0.93 ± 0.02 for Models 1, 2 and 3, respectively. The AUC was optimal for Model 3, and significantly differed between Models 3 and 1 (*p* = 0.027), and 3 and 2 (*p* = 0.029).

**Conclusions:**

Image features such as high or low uptake, symmetry or asymmetry, and comma- or dot-like profiles can be determined using ML. The diagnostic accuracy of differentiating PS/PD/DLB was the highest for the multivariate model with three features and age compared with the conventional ROI-based method.

**Supplementary Information:**

The online version contains supplementary material available at 10.1007/s12149-022-01759-z.

## Introduction

Iodine-123 [^123^I]-ioflupane is a radioactive marker of nigrostriatal neuron integrity. It has been clinically applied in Japan to differentiate parkinsonism with loss of dopaminergic terminals from that without nigrostriatal degeneration since 2014. Dopamine transporters scanned using ^123^I-ioflupane at an early stage have significant diagnostic specificity for clinically uncertain parkinsonian syndromes (PS) and Parkinson disease (PD) [1, 2] and good sensitivity for dementia with Lewy bodies (DLB) [3]. Criteria for abnormalities are based on the symmetricity of striatal uptake and reduced uptake in the putamen relative to the caudate nucleus. Count proportions of total striatal regions and non-striatal backgrounds (specific binding ratio; SBR), have revealed that normal and abnormal patients can be discriminated at an SBR threshold of ~ 4.5. The region-of-interest (ROI)-based method of calculating the SBR and asymmetry index incorporated in the DatView software (Nihon Medi Physics, Co. Ltd., Tokyo, Japan) is applied throughout Japan. However, the clinical diagnostic results of borderline uptake profiles are sometimes indeterminate. We, therefore, aimed to overcome the ambiguity of ROI-based methods using a machine learning (ML) approach to discriminate complex findings.

Machine learning (ML) is an artificial intelligence (AI) technology that has recently been applied in radiological and nuclear medicine studies [4–6]. Neural networks have also been applied to nuclear medicine studies of bone, and to myocardial perfusion images to identify bone metastases [7] and myocardial ischemia [8, 9], and they can be incorporated into routine clinical reports. As nuclear medicine functional images have relatively simpler accumulation profiles and generate less image data compared with other types of radiological imaging, AI algorithms incorporating various characteristics such as morphology, location, and radioactive distribution should have significant diagnostic value. However, only a few studies of ML applications have been described. Investigations are ongoing [10–12], and possible applications of AI are current topics in medical journals [4, 6, 13–15]. However, appropriate methods have remained undetermined, and their clinical use in nuclear medicine practice remains limited.

We, therefore, postulated that ML, such as nuclear medicine experts, could distinguish complex image features on ^123^I-ioflupane images and that multivariate models using ML-based features could improve diagnostic accuracy compared with conventional ROI-based indices.

## Methods

### Patients

We retrospectively analyzed ^123^I-ioflupane single-photon emission computed tomography (SPECT) images acquired from 239 patients at two hospitals. We determined image features of striatal uptake and appropriate methods for ML using consecutive imaging data from a municipal medical center (*n* = 137). We validated ML feature detection and created multivariable models for diagnosis in another consecutive data set (*n* = 102) from Kanazawa University Hospital. Patients with suspected neurodegenerative diseases and dementia including PD, PS and DLB were classified as having Parkinson syndrome or disease and DLB (PS/PD/DLB) or not. Table 1 shows the tentative diagnoses and the frequency of features in images from the municipal hospital. Neurologists at the University Hospital independently diagnosed Parkinsonism based on combinations of bradykinesia, resting tremor, rigidity, and posture. The PS/PD/DLB group (*n* = 65, 64%) comprised patients with PS (33%), PD (20%), and DLB (11%). The most likely diagnosis of PS (*n* = 31) was multiple system atrophy (MSA-C 6%, MSA-P 3%, not specified 1%), progressive supranuclear palsy (10%), corticobasal degeneration (5%), and not specified (6%). The group without PS/PD/DLB (*n* = 37, 36%) included patients with conditions, such as degenerative diseases with unknown etiology, drug-induced Parkinsonism, essential tremor, epilepsy, dystonia, and cerebrovascular disorders.

**Table 1:** Demographics of training-validation and test data sets

	Training—validation data set	Test data set
*N*	137	102
Age	75 ± 8	67 ± 15
Sex (male)	64 (47%)	43 (42%)
PS/PD/DLB	105 (77%)	65 (64%)
PD	35 (26%)	20 (20%)
PS	55 (40%)	34 (33%)
DLB	6 (4%)	11 (11%)
PD or PS	8 (6%)	0 (0%)
PD or DLB	1 (1%)	0 (0%)
Non-PS/PD/DLB	32 (23%)	37 (36%)
Alzheimer disease	1 (1%)	2 (2%)
Essential tremor	2 (2%)	4 (4%)
Other diseases and/or unknown etiology	29 (21%)	31 (30%)
*Image features*		
Low	106 (77%)	47 (46%)
Asymmetric	82 (60%)	38 (37%)
Dot-like	88 (64%)	48 (47%)
Abnormal	108 (79%)	42 (41%)

*DLB* dementia with Lewy bodies, *PD* Parkinson disease, *PS* Parkinson syndrome

### ^***123***^***I-ioflupane SPECT***

The SPECT images were acquired using Siemens ECAM SPECT (Kaga Medical Center) and GE Discovery NM/CT 670-Pro SPECT (Kanazawa University Hospital) cameras and low–medium- and extended low-energy general-purpose collimators, respectively. The images were acquired over a period of ~ 28 min that started from 4 h after an intravenous injection of ^123^I-ioflupane (DaTSCAN, 167 MBq). Camera rotation was continuous, with sampling in 3° steps, 3.5 s/view, four rotations. The matrix was 128 × 128 with 1.5× zoom, and the energy window for ^123^I was 159 keV ± 10%. Transaxial images were reconstructed using ordered subset expectation maximization (OSEM) and a Butterworth filter at 0.6 cycles/cm (order 5) for smoothing. X-ray CT-based attenuation correction was not applied.

### Data processing

**Fig. 1:**
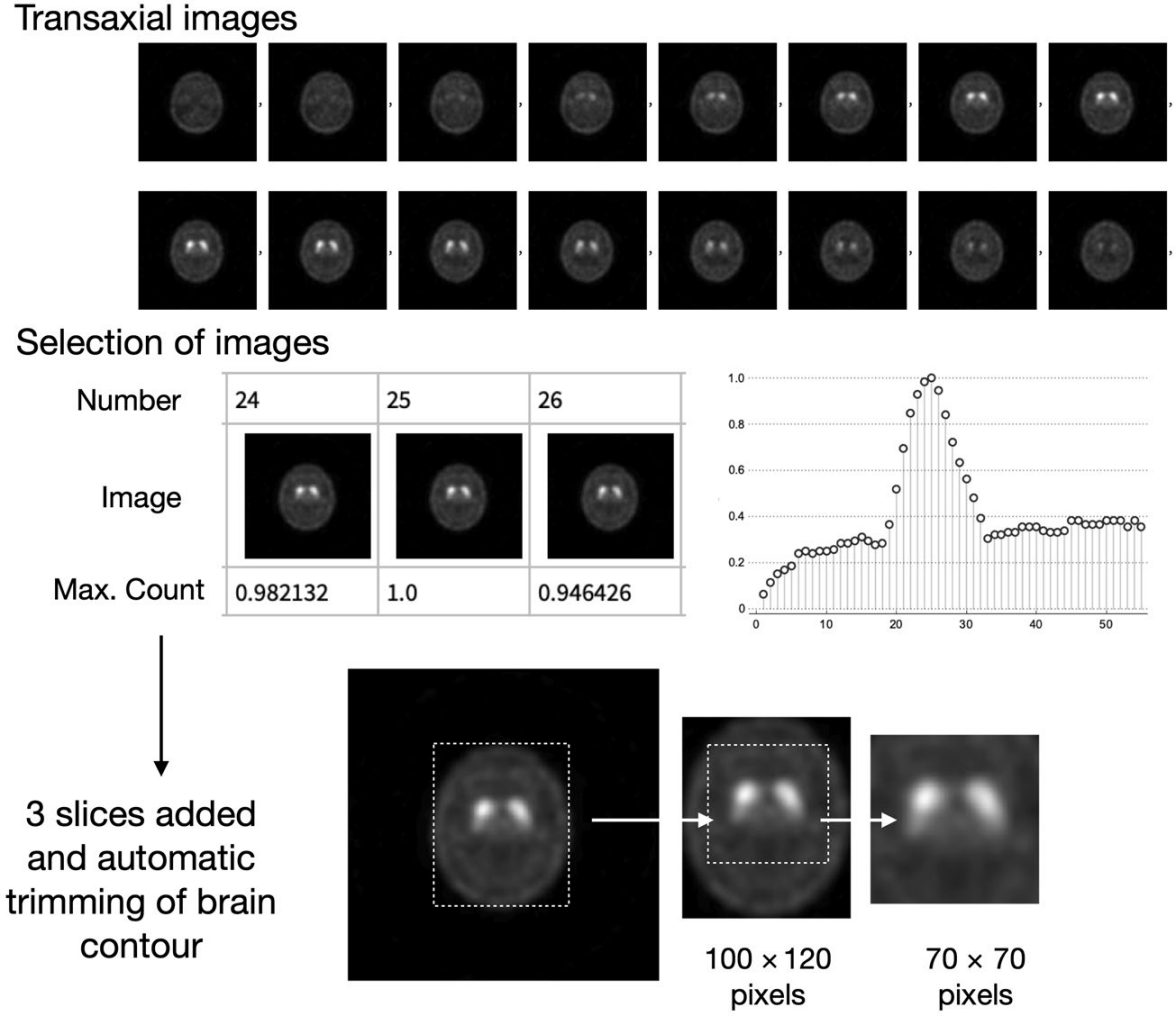
Processing data from DICOM image to automatic selection of striatal region

Transaxial images were prepared in an innominate Digital Imaging and Communications in Medicine (DICOM) format after standard reconstruction as indicated above. Figure 1 summarizes the processing method. Slices with maximal counts on the striatum were retrieved from all transaxial images, and three slices around the maximum count were added into a summed image. Background regions were removed from 128 × 128-matrix grayscale images, and brain contours were trimmed so that only the regions of a complete brain were included. The brain region was again modified to 100 × 120 pixels, then 70 × 70-pixel square images with striatal uptake on the center were automatically created. All pixel counts were adjusted based on maximum- and background-count normalized images (Fig. 1). The striatal region was determined by averaging all patients’ data and thresholding with a 30% cutoff, and the average background count was calculated by removing the striatal region in each patient. The maximum-count normalization used a range of 0.0–1.0, and background-count normalization was done to have background count of 0.15 (fixed value). We decided to use background-count normalized image for the training of ML after confirming the diagnostic metrics of both normalization methods.

### Image interpretation

**Fig. 2:**
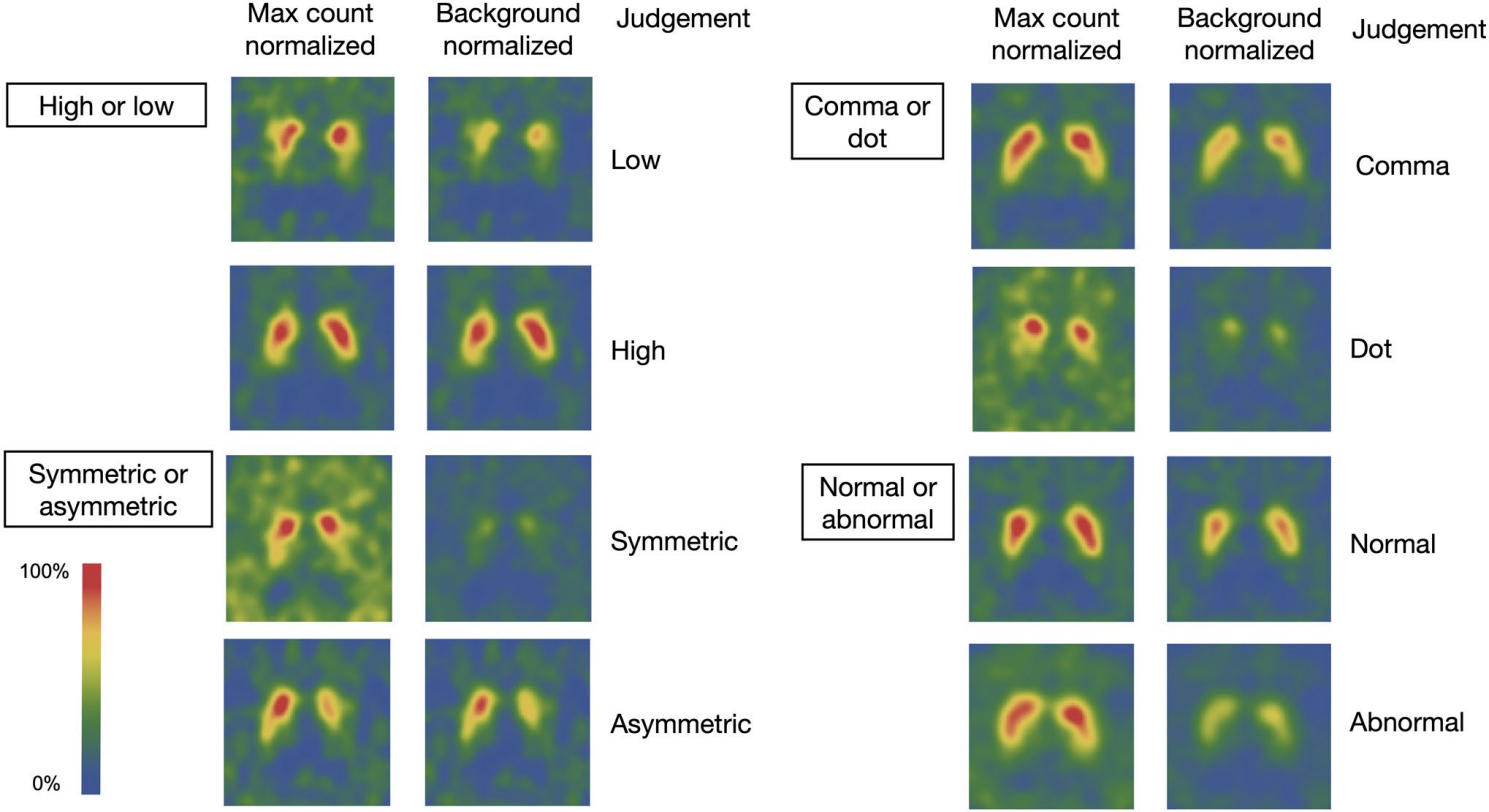
Determination of image features. Features of high or low, symmetric or asymmetric, and comma or dot, as well as overall impression of abnormality were determined

Three experienced nuclear medicine physicians reviewed the ^123^I-ioflupane images considering the basic status of the patients and reports. Image feature classification and overall impressions of abnormalities were confirmed by focusing on image profiles. The raw DICOM data of the images are shown in two display scales of the maximum- and background-count normalized images. Although the raw data were processed with grayscale images, subsequent rainbow colorization was added for display. Differing opinions among the three physicians were resolved by consensus (Fig. 2).

The following features of the images were evaluated: striatal uptake (high [normal] or low [feature 1, F1], symmetry (symmetry or asymmetric; feature 2, F2), striatum shape (comma-like or dot-like, respectively) determined by comparing activities in the caudate nucleus and putamen (feature 3, F3), and overall impression of the image (normal or abnormal; feature 4; F4). Visual interpretation integrating all information served as the ground truth of the image feature profiles during training. When differences in image profiles were obvious between brain hemispheres, the basis for a diagnosis was the profile on the worse side.

### Machine learning

Machine learning was conducted in the Wolfram Language (Mathematica version 12.3, Wolfram Research Inc., Champaign, IL, USA). The original 70 × 70 (4900) datapoints were directly used as input, and neither predetermined features nor statistical data were used. While ground truth of the training data set was image features determined by the nuclear medicine specialists, that of the test data set was the differential diagnosis of PS/PD/DLB and non-PS/PD/DLB determined by the neurologists. Ground truth interpretation and DICOM images were combined as “association” between judgements and images. The methods included gradient boosted trees (GBTs), logistic regression (LR), and k-nearest neighbors (KNNs). The internal parameters and hyperparameters of regularization and optimization, maximum training rounds, number and size of leaves, number of neighbors (*k* values) and smoothing variables were adjusted using the automatic setting for the Classify function [Wolfram Language]. To identify the optimal methods, 75% and 25% of the studies were, respectively, used for training and validation data sets (fourfold cross-validation by repeated tests). As horizontally flipped images were added to augment the number in the training database, the total numbers in the learning and test sets were, respectively, 137 × 75% × 2, and 137 × 25%.

Appropriate ML methods were applied to determine the metrics of recall (true positive rate), precision (positive predictive value), F1 scores (harmonic mean of precision and recall), and areas under receiver operating characteristics (ROC) curves (AUCs). The results of features F1–F4 were provided as probabilities of low uptake, asymmetry, dot-like shape, and abnormal image overall.

### ROI-based parameters

The SBR and the asymmetry index were calculated using DatView software. The SBR was the ratio of average counts between striatum and background regions proposed by Tossici-Bolt [13], which used pre-defined striatal ROIs and a non-striatal reference ROI to calculate right and left striatal uptake. The SBR was standardized for different camera conditions. The asymmetry index was the ratio of counts between right and left striatal uptake (%). Apart from the DatView indices, the average count ratio between the caudate nucleus and the putamen was calculated by setting circular and elliptical regions, respectively, as a fixed template [supporting data]. The locations and radii of these regions were manually adjusted to fit the shape of the striatum if necessary.

### Multivariable models for a diagnosis of PS/PD/DLB

**Fig. 3:**
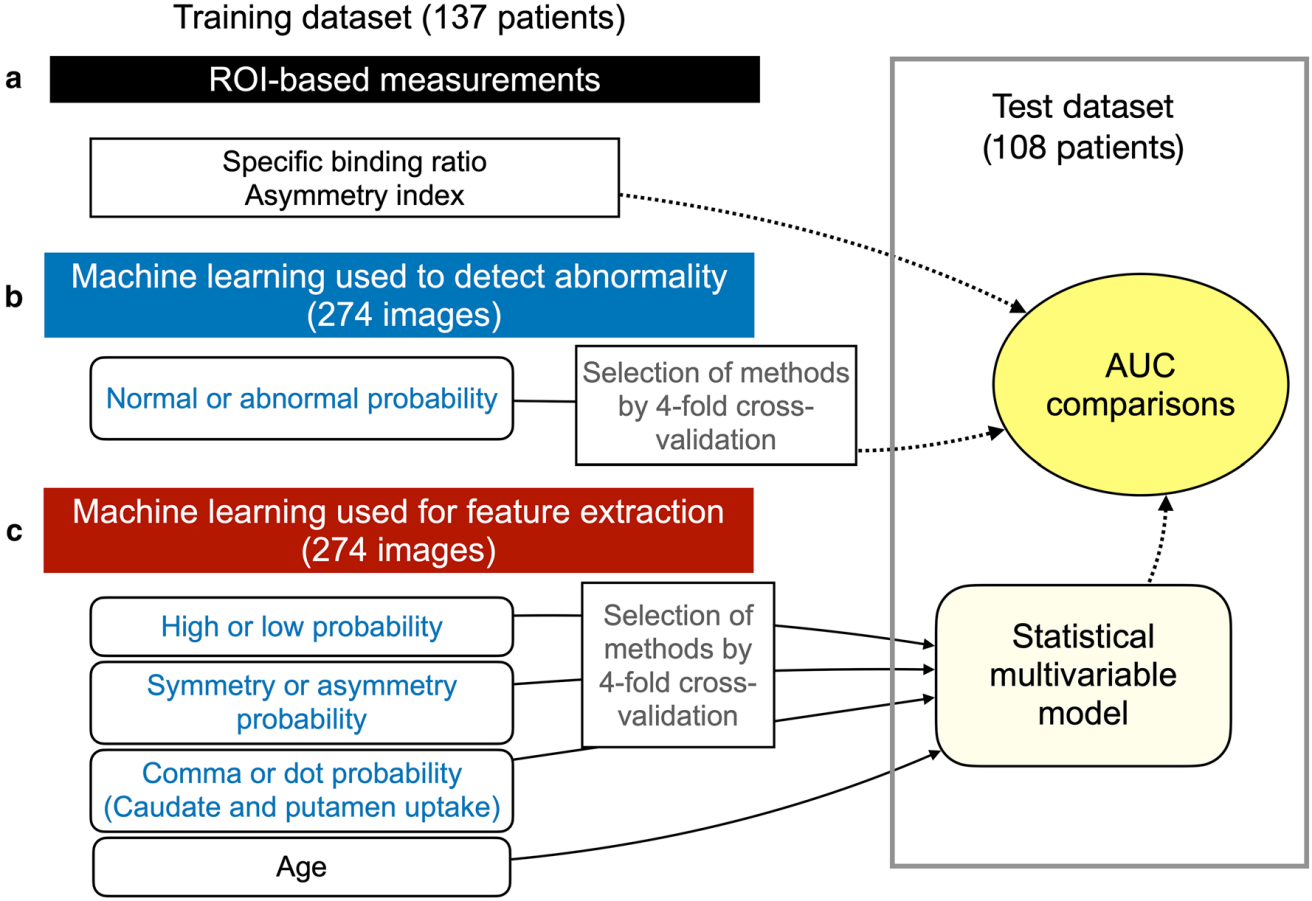
Three multivariable models. **A** Model 1: region of interest (ROI)-based calculation of specific binding ratio and asymmetry index. **B** Model 2: machine-learning-based direct judgement of abnormality. **C** Model 3: Logistic model combined with three features and age

We examined the features of the test data set (*n* = 102) to diagnose PS/PD/DLB by repeating the training with 137 patients (274 images including flipped images) using the ML methods. Figure 3 shows the three created and tested multivariable models. Model 1 included ROI-based SBR and an asymmetry index provided by DatView. Model 2 included the probability of abnormality (F4) derived from the best ML method. Model 3 included the ML-based probabilities of F1, F2, and F3 and patient age. The multivariable statistical Models 1 and 3 were assessed using logistic analysis and calculating probability using the formula, 1/(1 + Exp(− (*b*_0_ + Σ*b*_*i*_ × *x*_*i*_)), where *b*_0_ is an intercept and *b*_*i*_ is an estimate for the *i*th variable *x*_*i*_. The AUC was calculated for the three models to compare the diagnostic accuracy of PS/PD/DLB.

### Ethics approval

The Ethics Committee at Kanazawa University approved the study protocol, and the need for written informed consent from the patients was waived due to the retrospective collection of their data. The Ethics Committee at Kaga Medical Center approved collection of the innominate image data for ML. All included patients had the right to opt out of the study at any time.

### Statistical analysis

All results are expressed as means ± standard deviation (SD) or standard error (SE) as appropriate and compared using ANOVA and t tests. Asymmetrically distributed variables were examined using non-parametric Wilcoxon tests. The diagnostic metrics of recall, precision, *F*1 score, and AUC were calculated to compare ML methods. Diagnostic metrics for the PS/PD/DLB group are shown as AUCs for sensitivity and specificity. Threshold probability values corresponding to the highest sensitivity + specificity − 1 (Youden index) were determined. Combinations of variables in multivariable analyses were tested using a logistic model. A combined model with the highest AUC was created using the forward stepwise method. The density of feature probability was plotted using a violin-shaped density plot. The SBR and asymmetry index were calculated using DatView, and the SBR was adjusted for the camera and collimator at each institutional [16]. All data were statistically analyzed using Mathematica and/or JMP Pro v. 16 (SAS Institute, Cary, NC, USA). Values with *p* < 0.05 were considered significant.

## Results

### Fourfold cross-validation in the training data set

**Fig. 4:**
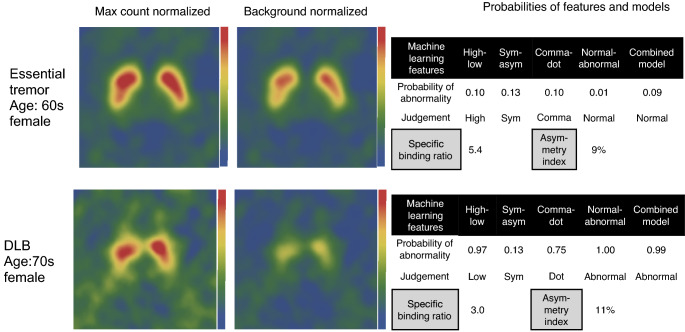
Patients with essential tremor and dementia with Lewy bodies (DLB) and image features. Probabilities were calculated by machine learning (ML) for low, asymmetry, dot-like, and abnormal features and based on a combined model using forward stepwise method

The fourfold cross-validation revealed that the high or low feature was equally identified by the LR, kNN and GBT methods (AUC 0.95–0.96, *p* < 0.0001 (Table 2). Symmetry or asymmetry was identified best by the kNN method (AUC 0.75), and the dot or comma-like feature was identified best by the GBT method (AUC 0.94) followed by the kNN (0.91) and LR (0.91) methods (*p* < 0.0001 for all) The ROI based indices showed AUC of 0.92 by SBR (*p* < 0.0001) for the high or low feature and 0.68 (*p* = 0.017) for the asymmetry index. The AUC of the putamen: caudate ratio was 0.85 (*p* < 0.0001). Figure 4 shows calculated probabilities for two patients.

**Table 2:** Fourfold cross-validation of training data set for feature detection (*n *= 137)

Features	AUC	Recall	Precision	F1 score	Accuracy	*p*
*High or low*						
LR	0.96	0.94	0.71	0.81	0.90	< 0.0001
kNN	0.96	0.84	0.79	0.81	0.91	< 0.0001
GBT	0.95	0.97	0.61	0.75	0.85	< 0.0001
SBR	0.92	0.97	0.64	0.77	0.87	< 0.0001
*Symmetry or asymmetry*						
LR	0.57	0.49	0.70	0.81	0.57	0.577
kNN	0.75	0.79	0.81	0.81	0.77	< 0.0001
GBT	0.54	0.18	0.79	0.81	0.48	0.059
Asymmetry index	0.68	0.83	0.75	0.81	0.72	0.017
*Comma or dot*						
LR	0.91	0.86	0.95	0.91	0.88	< 0.0001
kNN	0.91	0.88	0.90	0.89	0.85	< 0.0001
GBT	0.94	0.82	0.97	0.89	0.87	< 0.0001
PC ratio	0.85	0.74	0.92	0.82	0.79	< 0.0001
*Normal or abnormal*						
LR	0.91	0.86	0.95	0.91	0.88	< 0.0001
kNN	0.91	0.88	0.90	0.89	0.85	< 0.0001
GBT	0.94	0.82	0.97	0.89	0.87	< 0.0001

*GBT* gradient boosted tree, *kNN* k-nearest neighbor, *LR* logistic regression, *PC* putamen to caudate average count ratio, *SBR* specific binding ratio

### Identification of features in the test data set

The ability to detect features was examined in the test data sets after the training was competed using the 138 × 2 images (Table 3). Likewise, the AUC was good for the high or low feature in all methods, the kNN method had the best AUC for symmetry or asymmetry, and the GBT and LR methods had the best AUC for the comma or dot feature.

**Table 3:** Machine learning-based features and ROI-based parameters for feature detection in test data (*n* = 102)

	AUC	Recall	Precision	F1 score	Accuracy	*p*
*High or low*						
LR	0.95	1.00	0.67	0.80	0.85	< 0.0001
kNN	0.95	1.00	0.67	0.80	0.85	< 0.0001
GBT	0.94	0.90	0.77	0.83	0.89	< 0.0001
SBR	0.96	1.00	0.67	0.80	0.85	< 0.0001
*Symmetry or asymmetry*						
LR	0.58	0.53	0.76	0.63	0.63	0.202
kNN	0.68	0.62	0.77	0.69	0.67	0.003
GBT	0.67	0.57	0.79	0.66	0.66	0.001
Asymmetry index	0.64	0.95	0.71	0.81	0.75	< 0.0001
*Comma or dot*						
LR	0.92	0.73	0.98	0.84	0.81	< 0.0001
kNN	0.79	0.71	0.89	0.79	0.76	< 0.0001
GBT	0.88	0.83	0.93	0.88	0.84	< 0.0001
PC ratio	0.81	0.65	0.88	0.75	0.72	< 0.0001
*Normal or abnormal*						
LR	0.91	0.96	0.58	0.73	0.81	< 0.0001
kNN	0.89	1.00	0.57	0.72	0.80	< 0.0001
GBT	0.91	0.89	0.68	0.77	0.86	< 0.0001

*GBT* gradient boosted tree, *kNN* k-nearest neighbor, *LR* logistic regression, *PC* putamen to caudate average count ratio, *SBR* specific binding ratio

### Relationships between features and variables

**Fig. 5:**
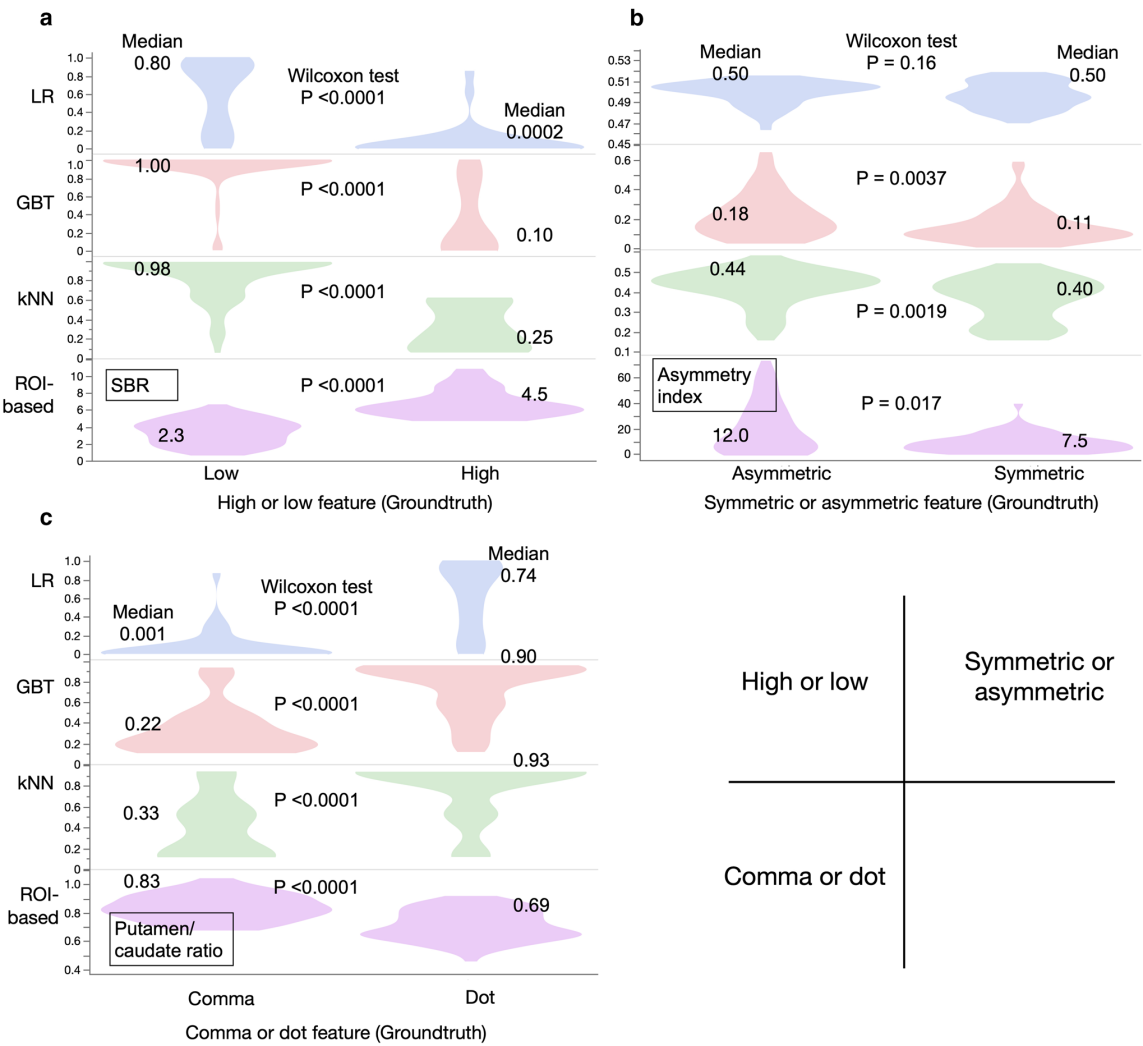
Density plots of probabilities of three features. Violin plots show density of medium values. *GBT* gradient boosted trees, *kNN* k-nearest neighbor, *LR* logistic regression

We examined relationships among feature groups, ML-based probabilities and ROI-based indices (Fig. 5). Density plots showed that the distribution of probabilities for the GBT and kNN methods had unique density shapes differed from conventional ROI-based methods. Fractions of probability near 0.0 and 1.0 were higher for ML-based features than ROI-based methods. In particular, the high or low and comma or dot features indicated that the discrimination capability was better for ML than ROI-based methods.

### Analysis of combinations of variables to identify PS/PD/DLB using AUC

**Fig. 6:**
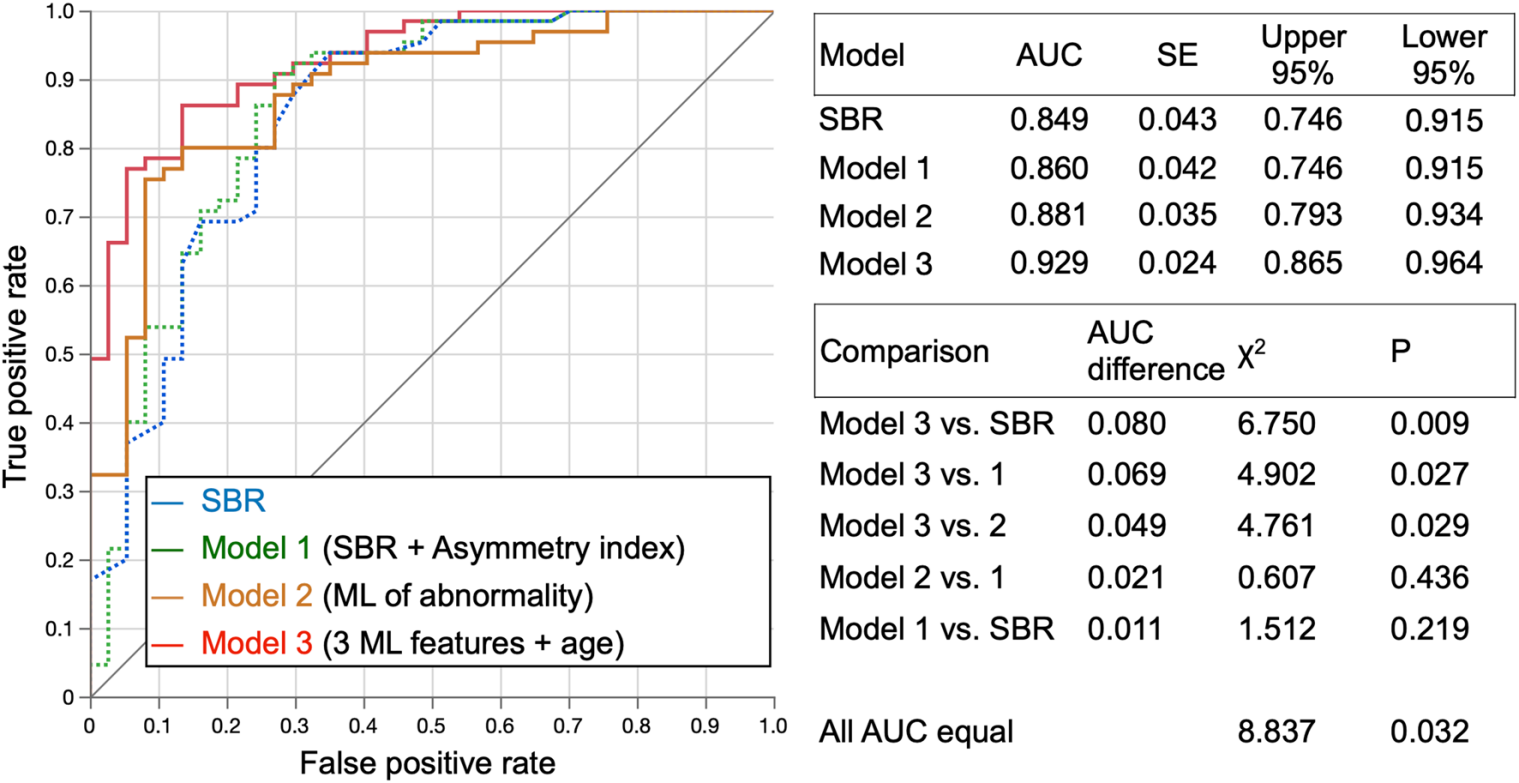
Receiver-operating characteristics (ROC) curves for Models 1, 2 and 3. *SBR* specific binding ratio

Table 4 and Fig. 6 summarize AUCs under various settings. The AUC was 0.85 (0.75–0.92) when PS/PD/DLB was diagnosed using the SBR and adding the asymmetry index only slightly increased it (Model 1, *p* = n. s.). The AUC was 0.88 (*p* = n. s. vs. ROI-based methods) when the ML-based abnormality (Model 2) was used, and adding the LR-based low and dot features and kNN-based asymmetry feature increased the AUC to 0.90 (*p* = 0.06 vs. SBR). Adding age to these features and selecting the variables with the best forward stepwise method (Model 3; combination of age + GBT-based low, kNN-based asymmetry, and LR-based dot features) increased the AUC to 0.93 (*p* = 0.009 vs. SBR; *p* = 0.027 vs. SBR and asymmetry index [Model 1]; *p* = 0.029 vs. Model 2). The cutoff probability value showing the highest sensitivity + specificity − 1 was 0.48 for SBR, 0.49 for Model 1, 0.84 for Model 2 (sensitivity/specificity = 89%/70% for cutoff 0.5), and 0.68 for Model 3 (sensitivity/specificity = 89%/73% for cutoff 0.5).

**Table 4:** Diagnosis of PS/PD/DLB based on features and combinations

	Method	AUC	Sensitivity	Specificity	*p*
*ROI-based methods*					
Specific binding ratio (SBR)		0.85	0.94	0.65	< 0.0001
Asymmetry index		0.77	0.54	0.95	< 0.0001
PC ratio		0.71	0.69	0.70	< 0.0001
SBR + asymmetry index (Model 1)		0.86	0.91	0.73	< 0.0001
SBR + asymmetry index + PC ratio		0.86	0.89	0.73	< 0.0001
*Machine learning-based feature*					
Normal or abnormal (Model 2)	LR	0.82	0.91	0.70	< 0.0001
	GBT	0.88	0.83	0.87	< 0.0001
*Combined model with ML features*					
Age + HiLo (LR) + SymAsym (kNN) + CommaDot (LR)		0.90	0.79	0.95	< 0.0001
Best forward-stepwise model: age + HiLo(GBT) + SymAsym (kNN) + CommaDot (LR) (Model 3)		0.93	0.86	0.92	< 0.0001

*GBT* gradient boosted trees, *HiLo* high/low, *kNN* k-nearest neighbors, *LR* logistic regression, *ML* machine learning, *PC* putamen to caudate average count ratio, *SBR* specific binding ratio, *SymAsym* symmetry or asymmetry ratio

## Discussion

Visual assessment of ^123^I-ioflupane is the first step of a diagnosis, and the present findings showed that multivariable models incorporating ML feature detection and age could distinguish characteristic abnormal findings with better diagnostic accuracy than conventional ROI-based quantitation.

### ***Abnormal visual image features with***^***123***^***I-ioflupane***

We used three features of striatal uptake for the ML. Visual assessment has played a vital role in the overall diagnostic process, since clinical ^123^I-ioflupane SPECT application started, even though semiquantitative software had already been created. The diagnostic criteria for abnormalities in the present and previous studies are similar. For example, a European study categorized image profiles of normal or almost normal putamen activity in one hemisphere with considerable changes on the other as type 1, greatly reduced uptake in the putamen on both sides as type 2, and virtually absent uptake as type 3 [3]. Moreover, visual scoring from 0 to 3 has also been introduced, in which normal uptake in all regions is scored as asymmetric activity (0), one putamen with reduced uptake (1), absent activity in the putamina of both hemispheres (2), and absent activity in the putamina of both hemispheres with greatly reduced activity in one or both caudate nuclei (3) [17]. Furthermore, similar distribution profiles have also been described, such as eagle-wing, almost normal symmetrical tracer with only a discrete tracer reduction in one or both putamina that resembles the shape of a wing; egg-shape, bilateral tracer reduction in the putamen and normal or almost normal findings in the caudate nucleus resulting in an oval shape, and burst striatum, a severe bilateral reduction with relatively high background activity [18]. Therefore, the image characteristics used herein, namely, low/high, asymmetry/symmetry, and dot-like/comma-like features, are basically in agreement with prior approaches.

### Semiquantitative methods

One prevalent semi-quantitative assessment method of ^123^I-ioflupane uptake is SBR, which is based on analyses of counts in the striatal regions of the two hemispheres and the background of the whole brain [13]. Although SBR evaluation is simple and widely used, cortical atrophy and dilated ventricles might result in background activity that is below the actual level. Even when the shapes of uptake on the caudate nucleus and putamen are asymmetric, average counts on striatal regions might still appear similar. In addition, when counts in both striatal regions are extremely reduced, the asymmetry indexes might be exaggerated, and the visual impression might depend on display scales and colorization. However, the laterality of accumulation profiles is basically interpreted as supporting a diagnosis of Parkinsonism if the predominant symptoms are contralateral. Therefore, using ML to identify asymmetry as accurately as visual interpretation by physicians might have practical clinical significance.

The correspondence of DaTQUANT software (GE Healthcare, Chicago, IL, USA) with expert reading based on a putamen-to-caudate ratio is 84% [19]. The intrinsic competence of the putamen-to-caudate ratio for an indication of neurological diseases has been proven [20]. However, the authors concluded that the considerable variability of this ratio might prevent it from being a reliable numeric marker for interpretating ^123^I-ioflupane images. This was partly due to difficulties separating the caudate nucleus from the putamen on SPECT images, and the nonuniformity of decreased uptake in the putamen and the caudate nucleus. In contrast, the ML adopted herein does not require strict ROI setting even in striatum with low uptake.

### Application of machine learning

We found that ML seemed to classify the clinically important features of striatal uptake and final impressions of normal and abnormal profiles. Machine-learning techniques have been applied to extract discriminatory features from patients with PS and PD. Shape analysis and surface-fitting-based features using a support vector machine were useful and superior to SBR-based analysis [21]. The data were augmented 44-fold, and neural network training achieved a final test AUC of 0.87 even in a small study of 54 normal persons and 54 patients with abnormalities [22]. A study in Japan in which a support vector machine was applied to ^123^I-ioflupane images and measured parameters based on volumes of interest found that quantitation improved and surpassed that of conventional SBR, the asymmetry index, and the putamen-to-caudate ratio [11].

We created a probability function for three diagnostic features and directly determined whether they were normal or not using twofold augmentation of the amount of data. The superior diagnostic accuracy provided by three features plus age was due to the direct determination of ML abnormalities and the application of characteristic abnormal profiles commonly included in the diagnostic process. We also found that the discrimination algorithm of ML significantly differed from ROI-based methods as shown in the probability density plot, indicating that AUC and ML-based approaches are complementary.

Since the development of a wide range of state-of-the-art technology is still underway, more analytical methods are needed for classifications and predictions. Current supervised learning methods include tree-based methods, such as decision trees, random forests, gradient boosted trees, regression models with a sigmoid activation function, such as logistic regression, and neural networks. Among these, we selected the LR, GBT and kNN methods for classifying most profiles of ^123^I-ioflupane images. While the performance of the kNN method was good for the asymmetry/symmetry feature, both the LR and GBT methods discriminated high or and dot- or comma-like features well. Combined assessment of characteristic features was also better than the direct classification of normal and abnormal profiles by ML. Adding age notably improved the ability to diagnose PS/PD/DLB. Since the SBR was needed considering the age-dependent decline in uptake [16], the improvement gained by combining age with image features was reasonable. The effect of age on SBR is significant, as SBR declines at a rate of 6.3% per decade. However, another study found no statistically significant age-related decline in SBR among adults aged > 60 years without Lewy body disease [23]. Combining ML features plus age is also convenient, because it provides explainable results from four variables that each contribute to the final diagnosis.

Finally, although the application of ML is generally considered promising, not one has yet been established as clinical diagnostic tool. This is partly due to limitations in versatile application to various image acquisition and reconstruction methods. Because sampling or selection bias of patients can be pitfalls when creating a good model, well-structured selection criteria are recommended. Although ML could help diagnosis, systematic checking by specialists will be required before it could be universally applied to clinical practice.

### Limitations

The total amount of information used for ML training is a limitation of this study. Although the results of the AUC were considered statistically significant, more data are desirable to enhance the reliability of image profile classifiers. While the data were acquired from two institutions under different conditions, SPECT reconstruction methods also vary among institutions. The influences of camera types, low- and low–medium energy collimators, CT-based attenuation correction, and high-resolution reconstruction should be further investigated for wider applicability of the algorithm. Although quantitative values are significantly influenced by these factors when using ^123^I tracers, such as metaiodobenzylguanidine [24], we suppose that the visual classification of abnormal profiles might not be significantly influenced. Considering effects of age, sex and factors of clinical signs and symptoms, more clinical information should be included from patients and healthy persons with various backgrounds to establish a more accurate diagnostic algorithm using ML.

## Conclusions

The ML method classified abnormal image features, such as low uptake, asymmetry, dot shapes, and abnormal profiles. The combined approach with three ML features and age was more accurate than the conventional ROI-based method and more practical from the viewpoint of the explaining the diagnosis. Approaches based on ML have added value from the perspective of conventional semiquantitative indices.

## Supplementary Information

Below is the link to the electronic supplementary material.

Supplementary file1 (PDF 682 KB)

## Abbreviations

AUC: Area under the ROC curve
DICOM: Digital Imaging and Communications in Medicine
DLB: Dementia with Lewy bodies
ML: Machine learning
PD: Parkinson disease
PS: Parkinson syndrome
ROC: Receiver operating characteristic
SBR: Specific binding ratio
SPECT: Single-photon emission computed tomography

## References

[1] Catafau AM, Tolosa E, Da TCUPSSG (2004). Impact of dopamine transporter SPECT using 123I-Ioflupane on diagnosis and management of patients with clinically uncertain Parkinsonian syndromes. Mov Disord.

[2] Tolosa E, Borght TV, Moreno E, Da TCUPSSG (2007). Accuracy of DaTSCAN (123I-Ioflupane) SPECT in diagnosis of patients with clinically uncertain parkinsonism: 2-year follow-up of an open-label study. Mov Disord.

[3] McKeith I, O'Brien J, Walker Z, Tatsch K, Booij J, Darcourt J (2007). Sensitivity and specificity of dopamine transporter imaging with 123I-FP-CIT SPECT in dementia with Lewy bodies: a phase III, multicentre study. Lancet Neurol.

[4] Al'Aref SJ, Anchouche K, Singh G, Slomka PJ, Kolli KK, Kumar A (2019). Clinical applications of machine learning in cardiovascular disease and its relevance to cardiac imaging. Eur Heart J.

[5] Kobayashi Y, Ishibashi M, Kobayashi H (2019). How will "democratization of artificial intelligence" change the future of radiologists?. Jpn J Radiol.

[6] Quer G, Arnaout R, Henne M, Arnaout R (2021). machine learning and the future of cardiovascular care: JACC state-of-the-art review. J Am Coll Cardiol.

[7] Nakajima K, Edenbrandt L, Mizokami A (2017). Bone scan index: A new biomarker of bone metastasis in patients with prostate cancer. Int J Urol.

[8] Nakajima K, Kudo T, Nakata T, Kiso K, Kasai T, Taniguchi Y (2017). Diagnostic accuracy of an artificial neural network compared with statistical quantitation of myocardial perfusion images: a Japanese multicenter study. Eur J Nucl Med Mol Imaging.

[9] Nakajima K, Okuda K, Watanabe S, Matsuo S, Kinuya S, Toth K (2018). Artificial neural network retrained to detect myocardial ischemia using a Japanese multicenter database. Ann Nucl Med.

[10] Magesh PR, Myloth RD, Tom RJ (2020). An explainable machine learning model for early detection of Parkinson's disease using LIME on DaTSCAN imagery. Comput Biol Med.

[11] Iwabuchi Y, Nakahara T, Kameyama M, Yamada Y, Hashimoto M, Matsusaka Y (2019). Impact of a combination of quantitative indices representing uptake intensity, shape, and asymmetry in DAT SPECT using machine learning: comparison of different volume of interest settings. EJNMMI Res.

[12] Tang J, Yang B, Adams MP, Shenkov NN, Klyuzhin IS, Fotouhi S (2019). Artificial neural network-based prediction of outcome in parkinson's disease patients using DaTscan SPECT imaging features. Mol Imaging Biol.

[13] Tossici-Bolt L, Hoffmann SM, Kemp PM, Mehta RL, Fleming JS (2006). Quantification of [123I]FP-CIT SPECT brain images: an accurate technique for measurement of the specific binding ratio. Eur J Nucl Med Mol Imaging.

[14] Otaki Y, Miller RJH, Slomka PJ (2022). The application of artificial intelligence in nuclear cardiology. Ann Nucl Med.

[15] Hirata K, Sugimori H, Fujima N, Toyonaga T, Kudo K (2022). Artificial intelligence for nuclear medicine in oncology. Ann Nucl Med.

[16] Matsuda H, Murata M, Mukai Y, Sako K, Ono H, Toyama H (2018). Japanese multicenter database of healthy controls for [123I]FP-CIT SPECT. Eur J Nucl Med Mol Imaging.

[17] Kane JPM, Roberts G, Petrides GS, Lloyd JJ, O'Brien JT, Thomas AJ (2019). (123)I-MIBG scintigraphy utility and cut-off value in a clinically representative dementia cohort. Parkinsonism Relat Disord.

[18] Sawada H, Orimo S (2019). Relationship between striatal 123I-FP-CIT uptake and cognitive functions in Parkinson’s disease [Japanese, Abstract in English]. Rinsho Shinkeigaku (Clin Neurol).

[19] Morbelli S, Arnaldi D, Cella E, Raffa S, Donegani MI, Capitanio S (2020). Striatal dopamine transporter SPECT quantification: head-to-head comparison between two three-dimensional automatic tools. EJNMMI Res.

[20] Matesan M, Gaddikeri S, Longfellow K, Miyaoka R, Elojeimy S, Elman S (2018). I-123 DaTscan SPECT brain imaging in Parkinsonian syndromes: utility of the putamen-to-caudate ratio. J Neuroimaging.

[21] Prashanth R, Roy SD, Mandal PK, Ghosh S (2017). High-accuracy classification of Parkinson's disease through shape analysis and surface fitting in ^123^I-ioflupane SPECT imaging. IEEE J Biomed Health Inform.

[22] Kim JS, Oh YS, Kim YI, Yang DW, Chung YA, You Ie R (2013). Combined use of 123I-metaiodobenzylguanidine (MIBG) scintigraphy and dopamine transporter (DAT) positron emission tomography (PET) predicts prognosis in drug-induced Parkinsonism (DIP): a 2-year follow-up study. Arch Gerontol Geriatr.

[23] Roberts G, Lloyd JJ, Petrides GS, Donaghy PC, Kane JPM, Durcan R (2019). 123I-FP-CIT striatal binding ratios do not decrease significantly with age in older adults. Ann Nucl Med.

[24] Nakajima K, Okuda K, Yoshimura M, Matsuo S, Wakabayashi H, Imanishi Y (2014). Multicenter cross-calibration of I-123 metaiodobenzylguanidine heart-to-mediastinum ratios to overcome camera-collimator variations. J Nucl Cardiol.

