# Supplementary material for: Diagnosis of Parkinson syndrome and Lewy-body disease using 123I-ioflupane images and a model with image features based on machine learning
Source: Ann Nucl Med. 2022 Jul 7;36(8):765–76. doi: 10.1007/s12149-022-01759-z (PMC9304062; doi:10.1007/s12149-022-01759-z)
Supplement: Supplementary file 1 — Supplementary file1 (PDF 682 KB) [file 12149_2022_1759_MOESM1_ESM.pdf]

Diagnosis of Parkinson syndrome and Lewy-body disease using ^{123}I-ioflupane images and a model with image features based on machine learning

Nakajima K, et al.

Supplementary data

Putamen and caudate uptake determined by ROI-based calculation. Sizes and shapes of circles and ellipses can be changed if necessary. Putamen: caudate average count ratio significantly differs between comma and dot-like patterns (n = 137).

Samples of regions of interest in caudate and putamen

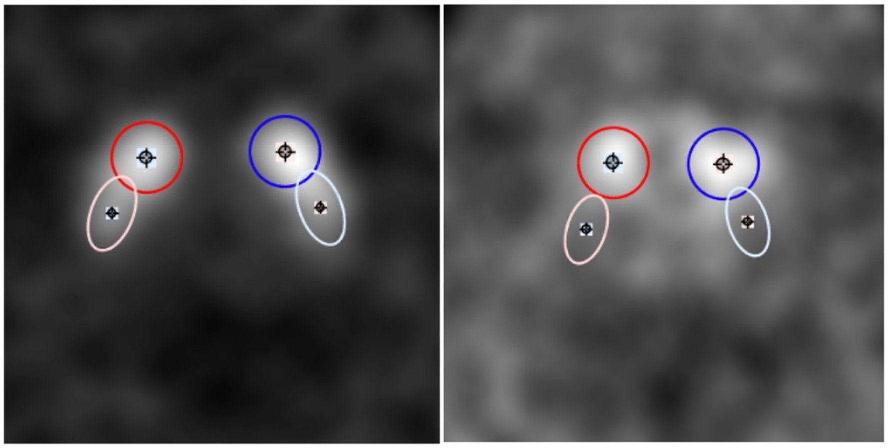

	right	left	ave
Caudate	0.676653	0.726538	
Putamen	0.475023	0.539423	
P/C ratio	0.702018	0.742457	0.722237

	right	left	ave
Caudate	0.759336	0.81927	
Putamen	0.455961	0.424251	
P/C ratio	0.600473	0.51784	0.559156

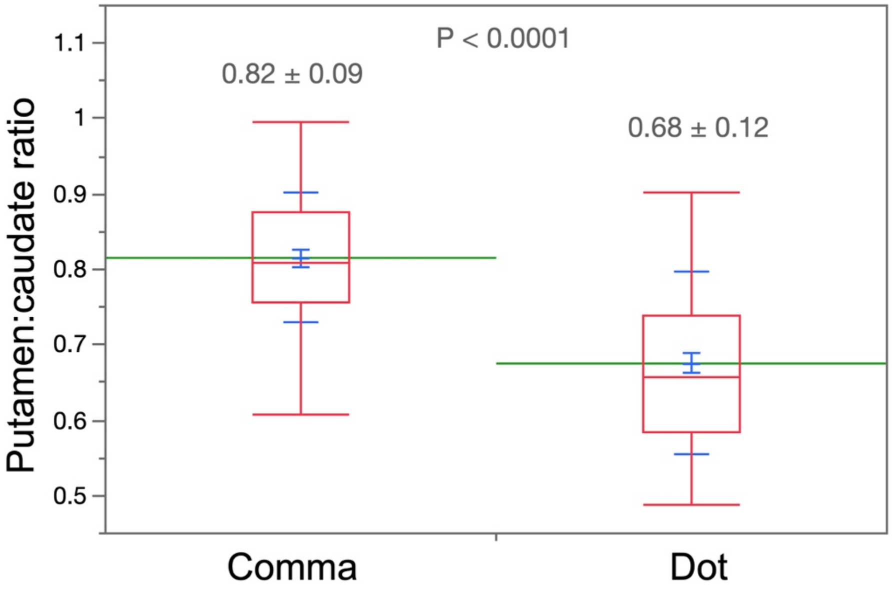

The boxplot denotes median and 1st and 3rd quartiles, and whiskers for value ranges.
Mean line (green), error bar and standard deviation (blue).
